# The Impact of the Quality of Nutrition and Lifestyle in the Reproductive Years of Women with PKU on the Long-Term Health of Their Children

**DOI:** 10.3390/nu14051021

**Published:** 2022-02-28

**Authors:** Maria Inês Gama, Alex Pinto, Anne Daly, Júlio César Rocha, Anita MacDonald

**Affiliations:** 1 Nutrition & Metabolism, NOVA Medical School, Faculdade de Ciências Médicas, Universidade Nova de Lisboa, Campo dos Mártires da Pátria 130, 1169-056 Lisboa, Portugal; inesrgama@gmail.com (M.I.G.); rochajc@nms.unl.pt (J.C.R.); 2 Birmingham Women’s and Children’s Hospital, Birmingham B4 6NH, UK; alex.pinto@nhs.net (A.P.); a.daly3@nhs.net (A.D.); 3 Reference Centre of Inherited Metabolic Diseases, Centro Hospitalar Universitário de Lisboa Central, 1169-045 Lisboa, Portugal; 4 CINTESIS—Center for Health Technology and Services Research, NOVA Medical School, Campo dos Mártires da Pátria 130, 1169-056 Lisboa, Portugal

**Keywords:** adherence, epigenetics, health, phenylketonuria, preconception, women

## Abstract

A woman’s nutritional status before and during pregnancy can affect the health of her progeny. Phenylketonuria (PKU), a rare disorder causing high blood and brain phenylalanine (Phe) concentrations, is associated with neurocognitive disability. Lifelong treatment is mainly dietetic with a Phe-restricted diet, supplemented with a low-Phe protein substitute. Treatment adherence commonly decreases in adolescence, with some adults ceasing dietary treatment. In maternal PKU, elevated blood Phe is harmful to the fetus so a strict Phe-restricted diet must be re-established preconception, and this is particularly difficult to achieve. A woman’s reproductive years introduces an opportunity to adopt healthier behaviours to prepare for successful pregnancies and positive health outcomes for both themselves and their children. Several factors can influence the health status of women with PKU. Political, socioeconomic, and individual food and lifestyle choices affect diet quality, metabolic control, and epigenetics, which then pre-condition the overall maternal health and long-term health of the child. Here, we reflect on a comprehensive approach to treatment and introduce practical recommendations to optimize the wellbeing of women with PKU and the resultant health of their children.

## 1. Introduction

Phenylketonuria (PKU, OMIM 261600) is an inherited metabolic disorder caused by mutations in the phenylalanine hydroxylase (PAH) enzyme that impairs phenylalanine (Phe) metabolism, leading to high blood and brain Phe concentrations. It is managed with a lifelong Phe-restricted diet and an adjunct pharmacological treatment, such as sapropterin or pegvaliase [1]. In maternal phenylketonuria (MPKU), it is established that Phe crosses the placenta’s blood membrane through a concentration gradient [2,3] and elevated blood Phe levels have a well-recognised teratogenic effect on the developing fetus, particularly in the early stages of pregnancy [4]. MPKU syndrome is characterized by foetal intrauterine growth retardation, facial dysmorphism, microcephaly, congenital heart disease, infant low birth weight, developmental delay, and intellectual disabilities [4]. There is also an increased risk of miscarriage, usually associated with poor maternal metabolic control [5]. Although there are several reports of pregnancy in women with PKU, little is known about the conception rates compared with the general population, though one recent UK/PKU study reported that 37% of 300 women aged ≥18 years had one or more children [6]. MPKU syndrome is preventable if women achieve rigorous blood Phe control by adhering to a Phe-restricted diet that is commenced preconception and continued throughout pregnancy. A considerable amount of professional health time and support is given to women during this challenging time.

In women with PKU, less consideration is given to the overall quality of nutritional care in the reproductive years (spanning from mid-adolescence until mid-adulthood) and interpregnancy. There is mounting evidence in all women of reproductive age that poor maternal and pregnancy health leads to a higher risk of disease in their children as they age [7]. The nutritional health of many women with PKU at the time of conception is likely to be sub-optimal, particularly if a strict dietary treatment has not been maintained through adult life. Some may have adopted an unhealthy eating pattern even if they are able to maintain optimal metabolic control. Furthermore, unplanned pregnancies at any point in time may increase the risk of nutrient imbalances. In England, 45% of all pregnancies are unplanned [7], and similar figures are observed in women with PKU, despite active health professional education to avoid unplanned pregnancy [5].

**Figure 1:**
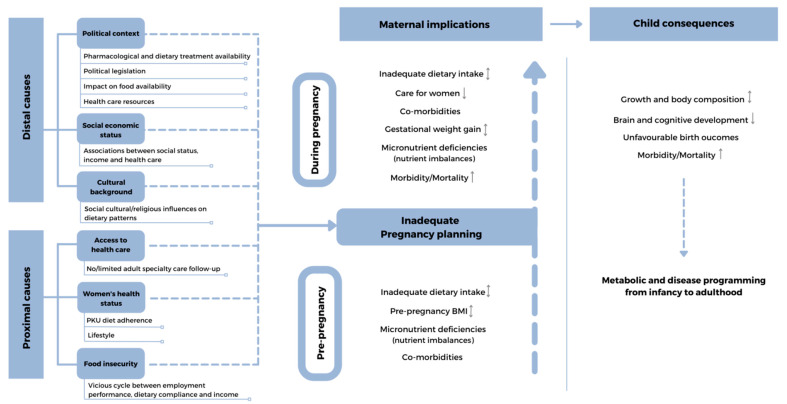
Preconception environmental and nutritional factors that may affect the foetal outcome in women with PKU. ↓-lower; ↑-higher; ↨-lower/higher; 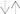 broken line-arrows-potentially lower/higher.

Therefore, the lifestyle choices of all women in reproductive years can have an enduring influence on the lifetime health of their children, and a clear focus on interventions before conception is necessary. Cohort studies have shown that improving dietary patterns for up to three years prior to conception can influence pregnancy outcomes, including lowering the risk of preterm birth [8]. Preconception environmental and nutritional factors that may affect the foetal outcome in women with PKU are presented in Figure 1. This review aims to highlight the importance of optimal nutrition, lifestyle, and environment in women with PKU in their reproductive years and offers proposals for pragmatic interventions that may improve the outcome of their children.

## 2. Nutritional Vulnerability of Women with PKU in Their Reproductive Years

### 2.1. Distal, Social, and Economic Causes of Nutritional Vulnerability in Adult Women with PKU

There are many economic and political factors that may lead to suboptimal nutritional outcomes associated with the availability of treatment for women with PKU. Health provision varies around the world, and some women with PKU have limited access to ‘free’ health care from public funding, while few hospitals provide PKU health care teams that provide expertise in the management of adult patients. Low Phe protein substitutes and special low protein foods (SLPFs) are an essential part of treatment but are expensive and may be unaffordable unless provided by insurance or state health care systems. Pharmaceutical treatments may be unavailable or even ineffectual (e.g., sapropterin) for adult patients with classical PKU without residual enzyme activity [4]. Many adult women may be unemployed, receive low earnings due to part-time work, or have minimal earning capacity due to impaired cognitive functioning, affecting their economic security, life quality, and ability to afford their dietary treatment. Political legislation that aims to improve the health of the entire population, e.g., food labelling laws and sugar taxes, may indirectly create additional treatment challenges because of further unintentional dietary restrictions for people with PKU.

Women who do adhere to dietary treatment are dependent on a Phe restricted diet and, if they have classical PKU, usually tolerate <500 mg/day Phe (equivalent to 10 g/natural protein) supplemented with protein substitutes. The protein substitutes are mainly comprised of Phe-free L-amino acids (AA) or low-Phe glycomacropeptide (GMP) and may potentially supply up to 80% of protein intake. Although they usually contain added tyrosine, micronutrients including vitamins, minerals, and long-chain fatty acids, such as docosahexaenoic acid (DHA), the lifetime outcome of habitually taking an artificial protein source is unknown. Amino acid supplements, compared with natural protein, are associated with less efficient utilization and early oxidation, and they may alter insulin release, glycaemic control, and endocrine regulation [9]. The impact on gut microbiota and long-term renal health is undetermined. SPLFs are high in carbohydrates [1,10,11] and contain isolated starches that are more refined or have a higher glycaemic index than equivalent foods made from wheat flour [12,13].

### 2.2. Proximal Causes Directly Related to Nutritional Vulnerability in Adult Women with PKU

Dietary adherence becomes increasingly challenging with age and metabolic control commonly deteriorates from adolescence [14,15,16,17,18]; it is estimated that 25% to 40% of adults who remain in clinical follow up discontinue treatment [19]. Most adults have difficultly re-establishing dietary control after a period ‘off diet’ or dietary relaxation [20]. Although more natural protein is consumed than prescribed, clinical practice suggests that the quality of foods eaten is poor, potentially leading to nutritional inadequacy [21,22]. Women may have a low IQ (associated with poor blood Phe control during childhood) and poor executive functioning and possibly have left home and lost the practical support of their parents. This affects their ability to self-manage a Phe restricted diet owing to the daily organisation and planning required [18,23]. Low mood or denial of the condition may also obstruct the ability of people to comply by reducing self-control or motivation. Poor knowledge of diet and food suitability, limited cooking skills and meal choices, the inability to read and interpret protein amounts on food labels, being unable to estimate protein exchanges, and difficulty accessing supplies of protein substitutes/SPLFs also influence the ability to adhere to the diet [24].

### 2.3. Health of Women with PKU

**Obesity**: The prevalence of overweight and obesity in all women of childbearing age is high, and approximately 39% of the world’s adult population is overweight, with 13% being obese [25]. Although a recent systematic review and meta-analysis of women with PKU [26] found that the body mass index (BMI) of patients with PKU was similar to their healthy controls, a subgroup of patients with classical PKU had a significantly higher BMI. The authors also noted a trend towards a higher BMI in females with PKU in all studies with male and female datasets. The BMI was also higher in an uncontrolled study in women with PKU, particularly if they had poor blood Phe control [27]. Adolescence is a critical period for the development of overweight and obesity [28], with a recent study illustrating that 28% (*n* = 101) of adolescents with PKU were overweight or obese [29].

**Eating disorders**: There is increasing evidence of eating disorders, food neophobia, and adverse attitudes towards food in adults with PKU [24,30,31,32]. Disordered eating refers to abnormal behaviours focused on eating or feeding, but it does not fit the pattern of a specific eating disorder [33]. It can manifest in restrictive, emotional, or uncontrolled eating. It is lower in severity and intensity than that of an eating disorder but impacts everyday life.

Fourteen percent of adults (*n*= 40/286) self-reported disordered eating in a survey reported by the UK National Society for PKU, with 4% receiving therapy for eating disorders. Individual patient stories described how they had an unpleasant relationship with food; others described how they used food as a reward [24]. Bilder et al. reported that 3.4% of patients (*n* = 128/3714) with PKU had an eating disorder compared with 0.9% in the general population [31]. Viau et al. discussed that 53% of adults (*n*= 9/18) on pegvaliase therapy had food neophobia with low enjoyment of food which did not appear to improve with a relaxed protein intake [32]. Luu et al. [33] found that in a group of adults with PKU (*n* = 15) aged 12–35 y, patients with poor metabolic control had symptoms of disordered eating at a higher frequency than those with good metabolic control. They were more likely to have been overweight, and there was an association between dieting and dissatisfaction with body image.

Food neophobia in adults with PKU may have its origins in childhood [34,35,36,37,38] and is likely to impede long-term dietary patterns, alter food selection, and lower nutritional quality later in life. Intransient feeding problems are very challenging to change, and diagnosing an eating disorder in a patient with PKU is difficult. Existing validated tools for the assessment of eating disorders may not be appropriate for individuals with PKU on a prescribed dietary treatment [33,39].

**Dietary pattern quality**: There are many concerns about the quality of diets consumed by women who have stopped dietary treatment, potentially causing nutritional fragility in reproductive years. Some patients remain on a self-imposed low-protein diet, avoiding protein-rich foods such as meat, fish, and milk for many years. If they eat higher protein foods, it is commonly only intermittently as many report guilt and having less food enjoyment if they eat foods contraindicated in their dietary treatment [24]. The discontinuation of a protein substitute, supplemented with vitamins and minerals, intensifies the risk of micronutrient deficiencies [18,22]. Women may have unpleasant memories of the taste, smell, and texture of protein substitute from childhood, or they may associate it with causing gastrointestinal symptoms such as reflux and constipation [24]. The absence of protein substitute intake may lead to the thinning of hair and poor skin condition associated with inadequate nutritional status [32]. There are reports of reduced or low normal serum urea levels [40]. In patients on a partial or minimal dietary treatment, a protein [41] and amino acid deficiency, particularly tyrosine [42] with low normal free carnitine values [43], are described.

Overall, there is little qualitative data discussing the dietary patterns of adults with PKU, and it is undetermined if they consume an adequate intake of fruit and vegetables. The habitual intake of meat, fish, dairy products, wholegrain cereals, and nuts and seeds is unknown but thought to be minimal. It is established that teenagers commonly eat high amounts of carbohydrates with a limited intake of fruit and vegetables [44], despite extensive dietary education.

**Figure 2:**
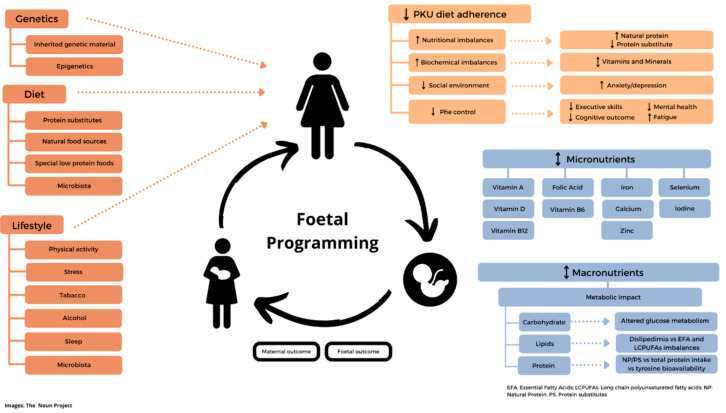
Foetal metabolic programming in women with PKU: influence of genetics, dietary management, and lifestyle on maternal and foetal outcome. ↓-lower; ↑-higher; ↨-lower/higher; 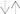 broken line-arrows-potentially lower/higher.

**Nutrient deficiency**: Women may be at particular risk of iron deficiency due to menstruation and the low intake of Phe-free/low-Phe protein substitutes. In a group of non-adherent UK adult patients with PKU (*n*= 14) who did not take protein substitute as prescribed, dietary intakes of iron, zinc, vitamin D3, magnesium, calcium, selenium, iodine, vitamin C, vitamin A, and copper were significantly lower than adherent patients (*n* = 16) and were below the UK Reference Nutrient Intakes [21]. Rohde et al. demonstrated that in 67 patients with PKU who consumed a ≤0.5 g/kg protein equivalent from a protein substitute that calcium and vitamin D intake was low, and the majority had low plasma 25-OH- vitamin D levels [22]. Vitamin B12 [41,45], zinc [21,46], and selenium [21,41,47] inadequacies are also reported in adult patients. Lower dietary adherence is associated with mild iodine deficiency and lower urinary selenium levels [48]. Pregnancy also increases the requirements for several macro- and micro-nutrients, compounding the risk of nutritional imbalance in women. The influence on maternal and foetal outcome of genetics, foetal programing, dietary management, and lifestyle of women with PKU are presented in Figure 2.

### 2.4. Nutrition, Foetal Metabolic Programming, and Epigenetics

The foetal programming concept suggests that maternal nutritional imbalance may have a persistent effect on the health of their children. It may pre-condition for metabolic syndrome and lead to long-term, irreversible changes in the organs and metabolism [49]. Poor maternal nutrition has been linked with early embryogenesis and foetal growth abnormalities, cardiovascular disease risk, and metabolic and renal dysfunction [50,51]. The Dutch famine studies clearly demonstrated how poor nutritional intake affects foetal outcomes. Children from pregnancies influenced by famine in early gestation had increased disease and metabolic risk in adulthood [52]. Even second-generation children of women who experienced famine in pregnancy were at increased metabolic risk, creating a transgenerational effect. Foetal epigenetic programming could play a key role in foetal metabolic programming [53,54].

Epigenetics is defined as changes that modify gene expression and cellular function; they do not change the DNA nucleotide sequence. Unlike genetic changes, these are reversible [53,55]. Epigenetic changes occur when environmental conditions, such as malnutrition or stress during critical periods in early life, modify metabolic and developmental pathways, in turn leading to alterations in their function [55,56,57] and the predisposition of individuals to disease in later adulthood [58]. Barker [59] first suggested that environmental events occurring during pregnancy could have consequences in adult life, leading to cardiometabolic disease. Thus, the quality of nutrition and nutritional imbalances, dietary restriction, eating behaviors, lifestyle, and nutritional supplementation may affect nutritional programming before, during, and between maternal PKU pregnancies [49,57,60,61].

Micronutrients, including iron, zinc, folic acid, and other vitamins, contribute to epigenetic modifications during organogenesis in early pregnancy [58,62]. Methyl-donor groups, such as folate and vitamin B12, are vital for embryo and early foetal development [62]. Preconception zinc deficiency compromises foetal and placental growth and neural tube closure [63]. Folate, vitamin B12, methionine, choline, and betaine can affect DNA methylation and histone methylation. Folic acid, vitamin B12, and zinc participate in brain DNA and RNA synthesis, which begins early in gestation. Decreased vitamin B12 in the first trimester, associated with raised levels of folate, predicts increased central obesity and insulin resistance in the offspring [62]. Vitamin B12 has also been shown to affect myelination, which begins during gestation, and may affect cognitive functioning.

Folic acid and vitamin B12 participate in the folate–methionine cycle [64]. They are essential in the remethylation of homocysteine into methionine, which, consequently, generates S-adenosylmethionine, a methyl-donor molecule and folic acid essential in the prevention of neural tube defects (NTDs) [65]. There is evidence of inadequate intakes of folate and vitamin B12 in adult patients with PKU [41,66,67,68]. Many countries have a folic acid food fortification policy to decrease the incidence of NTDs or recommend folic acid supplementation during preconception and early pregnancy. However, regular foods fortified with folic acid (e.g., bread, pasta, and flour) are unsuitable for people with PKU. Protein substitutes are supplemented with folic acid, but reports of inadequate folic acid intake are described in non-adherent adults. In women with PKU, 400 µg/day of folic acid supplementation is recommended during preconception and the first 12 weeks of gestation [4]. Vitamin B12 is obtained from animal foods, which are excluded in a Phe-restricted diet, and acceptable intake is usually only associated with adherence to a nutritionally fortified protein substitute.

There is also evidence from animal and clinical studies that maternal overnutrition can lead to epigenetically mediated alterations in different physiological homeostatic regulatory systems and is associated with increases in the cardiometabolic risk in infants [56]. Observational evidence suggests that metabolic changes due to parental overweight/obesity affect epigenetic markers in oocytes and sperm alike and may influence epigenetic programming and reprogramming processes during embryogenesis [69]. However, mechanisms underlying overweight development and foetal adipogenic programming through influences of early-life stages are still poorly understood.

### 2.5. Role of Key Micronutrients in Reproductive Nutrition

**Iron**: A major public health problem that affects all women of reproductive age is anaemia, and in 2019 the global prevalence of anaemia in women of reproductive age (15–49 years) was 29.9% [70]. Anaemia has been associated with an increased risk of poor birth outcomes (low birth weight, preterm births, being small for gestational age, stillbirth, and perinatal and neonatal mortality) and adverse maternal outcomes (maternal mortality, postpartum haemorrhaging, and preeclampsia [71,72]. Perinatal iron deficiency is associated with long-term cognitive abnormalities as iron plays an important role in normal neurodevelopment through enzymes controlling neurotransmitter synthesis, cell division, neuronal energy metabolism, and myelination [73].

Preconception iron status is critical [65], and in women with PKU, the main sources are protein substitutes; women are particularly at risk of deficiency if adherence to this nutrition source is low. Several studies have reported an inadequate micronutrient status, including iron, particularly in non-adherent patients [21,22,74]. Green et al. identified that off-diet individuals with PKU with a blood Phe ≥600 µmol/L had iron intakes below the country-specific recommendations [74]. In a further two studies, patients with PKU who had stopped dietary treatment had significantly lower iron intake compared to adherent patients [21,22].

**Iodine**: Iodine is important in early foetal development and is associated with its involvement in thyroid function and foetal brain development [65]. Due to an increase in the iodine requirement for brain development in early pregnancy, iodine deficiency in the preconception period increases the risk of developmental delay in a child [65]. A meta-analysis by Levie et al. showed that a lower urinary iodine-to-creatine ratio during pregnancy was associated with a lower verbal IQ [75]. In women with PKU, iodine status is strongly influenced by a dietary adherence to protein substitutes supplemented with micronutrients, the main dietary source of iodine [21,22,48,74,76].

**Zinc:** In an in vivo model, acute dietary zinc deficiency before conception compromised oocyte epigenetic programming and disrupted embryonic development [77]. It is also important for immune function, foetal growth and neurological development, and potentially lowers the risk of preterm birth [65]. Low zinc intakes are commonly observed in women with PKU [21,74].

**Long-chain polyunsaturated fatty acids (LC-PUFAs)**: These play an important role in the inflammatory response as eicosanoid precursors, as well as an important role in foetal–infant brain development in the later stage of pregnancy and early infancy. It is crucial that adequate maternal LC-PUFAs reserves are maintained early in pregnancy and for foetal use in later stages of development [78]. The placenta relies on fatty acids as a major energy source and disturbances in nutritional status could cause placental dysfunction, such as angiogenesis occurring in the first trimester and, consequently, compromise of foetal development [78].

The placental transport of LC-PUFAs is altered in maternal obesity and diabetes, which consequently has implications for foetal metabolic status [78]. Low DHA concentrations are reported in patients with PKU and during pregnancy [79,80,81,82] if women do not receive a supply from a protein substitute supplemented with DHA. Pregnant women should be supplemented with an additional supply of ≥200 mg DHA/day, over and above the intake recommended for an adult’s general health, and usually achieves a total intake of ≥300 mg DHA/day [83]. This should be given to all women with PKU considering pregnancy and throughout pregnancy [4,83].

**Over-nutrition:** Obesity is associated with an increased risk of most major adverse maternal and perinatal outcomes, including infertility, miscarriages, complications during pregnancy (pre-eclampsia and gestational diabetes) and delivery (macrosomia), congenital anomalies, stillbirth, unsuccessful breastfeeding, and even maternal death [65,84,85,86,87,88]. A higher BMI before pregnancy is associated with a more significant fat mass gain during pregnancy and is correlated with fat retention postpartum. It is also a strong predictor for increased birth weight, as well as for childhood overweight and obesity [69].

Obesity in pregnancy has been shown to significantly alter glucose metabolism leading to impaired fasting glucose reduction in early pregnancy and a considerable increase of peripheral and hepatic insulin resistance [56]. Any obesity-related, pre-pregnancy insulin resistance is associated with an increase of gestational diabetes and, consequently, a higher risk of foetal glucose metabolism impairment, hyperinsulinemia, and type 2 diabetes.

**Maternal gut microbiome**: Maternal health and diet play a critical role in the foundation of a child’s gut microbiome with long-lasting health implications. The rise in oestrogen and progesterone during pregnancy alters the gut function and microbiome composition, increasing vulnerability to pathogens. Throughout pregnancy, the gut microbiota progressively changes, with the greatest change occurring in the ratio of specific key bacteria (e.g., Firmicutes/Bacteroidetes ratio) mimicking the higher levels of Firmicutes seen in obesity [89]. Gut microbiota [90] can interact and be modulated by dietary factors. Prebiotics, such as fructooligosaccharides and galactooligosaccharides, have a positive influence on the gut microbiota composition. Little is known about the carbohydrate intake of adults with PKU. In a Phe-restricted diet, many of the carbohydrate sources allowed are based on simple sugars, e.g., sucrose and fructose, and this may cause rapid deregulation in the composition of the gut microbiota and, hence, metabolic dysfunction in the host [91]. Although some SPLFS contain added fibre, it is usually in the form of hydrocolloids to help their structure rather than provide nutritional benefits [11,44]. There is evidence that patients with PKU may have dysbiosis with less variety of bacteria, which may interfere with an optimal metabolism [92]. As well as the quality of carbohydrate intake, the high consumption of snacks, late-night eating, and skipping breakfast can also affect the gut microbiota composition [91].

**Sleep hygiene**: Sleep patterns may be disturbed in adult patients with PKU [93]. Quantity and quality of sleep play important roles in metabolic regulation and homeostasis [57,94]. A good night’s sleep is associated with improved glucose, lipid, and energy metabolism, cardiovascular risk, inflammatory response, neurocognitive function, and mental health status [94,95].

### 2.6. Interventions to Improve Nutritional Health in the Reproductive Years of Women with PKU

Preconception care has been defined as ‘‘any intervention provided to women of childbearing age, regardless of pregnancy status or desire, before pregnancy, to improve health outcomes for women, newborns and children’’ [96]. In MPKU, it is important to identify any opportunities for improving nutrition prior to pregnancy using evidence informed interventions. It should be accepted that improving women’s nutritional status may take several years and may be particularly challenging to maintain due to the high levels of food neophobia, maladaptive feeding behaviours, and limited food choices. In addition, individual motivations to engage with improving preconception nutrition will differ according to age, mental health, cognitive ability, and executive function. Understanding and harnessing these motivations will be key to successful intervention. Interventions to improve the nutritional status of PKU patients during their reproductive years are presented in Table 1.

## 3. Conclusions

The health of a mother and her children cannot be completely separated, and a heightened awareness of the importance of preconception health, particularly diet and nutrition, is essential in women with PKU. Birth outcomes are influenced by the long-term interaction of a woman’s biology, behaviour, social and environmental factors, and quality of diet. Therefore, the optimal health status of women with PKU before and inter-conception is essential. It is important that there is attention to dietary adequacy, healthy weight, and lifestyle. Women should be encouraged to maintain dietary and pharmaceutical treatments for PKU for optimal neuropsychological functioning and the provision of self-care during their reproductive years. In addition, the attainment of optimal nutrition should be the goal of health professionals. Any approach that improves the long-term nutritional health of women with PKU will help enhance the well-being of their future children.

## Figures and Tables

**Table 1:** Interventions to improve nutritional health in women with PKU in their reproductive years.

Intervention	Recommendation/Action by Individual Women with PKU or Health Care Teams
Prevention of overweightand obesity	Substantial weight loss is particularly difficult in women with PKU due to the catabolic effect of lowering energy intake on PKU and impact on metabolic control and may take months and even years to achieve. Ideally, healthy weight should be established before or during adolescence and pre-pregnancy. Undertake regular preconception assessments of weight, BMI, nutritional monitoring, dietary patterns/intake, and lifestyle. Women with PKU should try and maintain an adequate balance between energy intake and expenditure. Increase amount and range of fruits, vegetables, and plant foods whilst limiting the intake of total fats, free sugars, and sodium. Decrease snacks and late-night eating. Encourage breakfast. Reduce sedentary activity such as television or computer viewing.
Regular exercise	Higher levels of preconception physical activity are associated with a lower risk of gestational diabetes and pre-eclampsia [97]. Address sedentary lifestyles early in life by promoting physical activity. Encourage 10,000 steps daily of unstructured activity in the light-to-moderate intensity range that are usually part of daily living (e.g., cycling, climbing stairs, and walking). Sports and structured activities: encourage 150 min per week of structured activities (that range from a moderate to vigorous intensity) [97]. Pedometers or similar apps can be used as forms of motivational support.
Improve quality of Phe-restricted diet	Promote adherence to dietary treatment and explore individual resistance to maintaining a Phe-restricted diet. Promote dietary diversification within the limits of dietary restriction. Encourage at least 400 g/day of fruit and vegetables, equivalent to 5/daily portions [98]. A range of different fruits and vegetables will provide different nutrients, phytochemicals, and fibre [90]. The EFSA recommends 25 g/day of fibre [99]. To help achieve this, a high intake of fruit and vegetables and wholegrain cereals (within natural protein allowance) is necessary. Focus on fat quality rather than quantity; monounsaturated and polyunsaturated fat sources provide health benefits associated with triglyceride and cholesterol metabolism. Avoid trans fats and lower saturated fat intake [90]. Give careful guidance on the choice of SLPF’s as some may contain increased amounts of saturated and trans fats when compared to regular foods [11]. Ensure an adequate intake of essential fatty acids such as omega-3 and omega-6, with an emphasis on the optimal ratio of omega-3/omega-6. Encourage less added salt at the meal table and in cooking. Replace salt with herbs and spices. Provide social support to adults with PKU to help attain financial assistance to help purchase basic foods.
Encourage a healthy gut/gut microbiota	Assess gut health (particularly check for presence of gastro-intestinal reflux and constipation) at least annually. Fibre sources, including fruit and vegetables, augment microbiota diversity and are beneficial for gut health [90]. Fibre fermentation end-products and short-chain fatty acids (SCFA) have a role in preventing gut dysbiosis associated with metabolic dysfunction and immune response. SCFA, acetate, propionate, and butyrate are important modulators of gut microbiota [100]. Probiotic foods or supplements may offer additional protection. No controlled supplement trials of probiotics have been conducted in women with PKU.
Ensure a vitamin/mineral enriched protein substitute is taken in prescribed amounts	Explore any patient barriers to taking a protein substitute as prescribed. Give protein substitute in at least 3/daily doses and spread evenly throughout the day to minimise blood Phe fluctuations and to aid bioavailability of nutrient absorption. Protein substitutes help ensure that many macro- and micro-nutrient requirements are met. Meta-analyses confirm that supplementation or fortification with the ‘big four’ micronutrients (vitamin A, iron, zinc, and iodine) is efficacious to reduce the risk of infectious disease and improves growth and cognitive outcome in infants.
Give nutrition supplementsin the peri-conceptual period	Give 400 mg/day of folic acid in the periconceptual period to reduce the risk of neural tube defects by up to 72% [4,101]. Folic acid supplementation will also decrease the risk of pre-eclampsia, miscarriage, low-birth weight, being small for gestational age, a stillbirth, neonatal death, and autism in children [61,102]. A minimum of 4–6 weeks of folic acid supplementation is required to reach adequate levels before neurulation begins three weeks after conception. There is no information about adherence with folic acid supplementation in women with MPKU.
General lifestyle factors	Discourage smoking. While there are no published trials showing that reducing smoking before conception improves outcomes, indirect evidence suggests that smoke-free legislation in different countries has been associated with substantial reductions in preterm births [8]. Encourage moderate alcohol consumption in case of unplanned pregnancy. Maternal alcohol consumption can result in a range of foetal alcohol spectrum disorders [8]. Evaluate sources and perceived levels of stress, mood, and support systems. Offer psychological support and counselling. Encourage attendance of ‘online’ group mindfulness/support sessions.
Use of sapropterin	Sapropterin can liberate a woman’s diet and increase natural food sources and nutrient intake in sub-groups of responsive women, but education and careful monitoring is needed, as changes in food patterns may have a negative impact on nutrient adequacy [32,103].
Maintain regular nutritional monitoring	Monitor nutritional intake at each dietetic review. Assess food patterns and check for any disordered eating or maladaptive eating practices. Monitor weight, BMI, and abdominal circumference at each face-to-face review. Review the condition of the hair, skin, and nails. Assess patients’ biochemical nutritional status at least once a year. Assess for risk of comorbidities by monitoring lipid profile, blood pressure, and HbA1c [104]. Monitor blood Phe levels (according to European PKU guidelines) [4].
Encourage good sleep hygiene	Evaluate sleep patterns. Adults should aim to sleep at least 7 h per night to maintain optimal health [105]. Eating at late hours in the day has a negative effect on glucose, lipid, and energy metabolism [94], although a late-night dose of protein substitute may help decrease Phe fluctuations.

Abbreviations: PKU, Phenylketonuria; MPKU, Maternal Phenylketonuria; BMI, Body mass index; HbA1c, hemoglobin A1c; EFSA, European Food Safety Authority; Phe, Phenylalanine.

## Data Availability

Not applicable.
